# Anomalous Aortic Origin of the Right Coronary Artery Between the Right and Left Coronary Sinuses: A Case Report

**DOI:** 10.7759/cureus.99727

**Published:** 2025-12-20

**Authors:** Maria Eduarda Costa, Catarina Bettencourt, Joana Guardado, Tânia Virginia Tavares dos Santos, Andreia Vieira da Cruz

**Affiliations:** 1 Family and Community Medicine, Buarcos Family Health Unit, Baixo Mondego Local Health Unit, Figueira da Foz, PRT; 2 Cardiology, Figueira da Foz District Hospital, Baixo Mondego Local Health Unit, Figueira da Foz, PRT

**Keywords:** cardiac ct angiography, coronary artery anomalies, early diagnosis, interarterial course, myocardial ischemia, primary care, right coronary artery, sudden cardiac death, unroofing surgery

## Abstract

Anomalous aortic origin of the right coronary artery (R-ACAOS) is a rare congenital anomaly associated with an increased risk of myocardial ischemia and, in some cases, sudden cardiac death, particularly when the anomalous artery follows an interarterial course between the aorta and the pulmonary artery. This report presents the case of a 71-year-old man with nocturnal palpitations and atypical chest discomfort as the main symptoms, without syncope. Holter monitoring revealed first- and second-degree atrioventricular (AV) block. Coronary computed tomography angiography (CCTA) identified R-ACAOS with a short interarterial trajectory between the aorta and pulmonary arteries, with no significant ostial narrowing or other high-risk anatomical features. Cardiac magnetic resonance (CMR) demonstrated inferior wall ischemia.

The patient was referred to the cardiothoracic surgery department, where surgical unroofing was planned to eliminate potential dynamic compression and achieve complete symptom resolution. This case underscores the importance of prompt recognition of R-ACAOS, even in elderly patients, and highlights the crucial role of coordinated care between primary and specialized services. Comprehensive anatomical evaluation using CCTA or cardiac MRI is essential to assess risk and guide appropriate management. Surgical unroofing remains a reliable and effective intervention for symptomatic patients with interarterial R-ACAOS; thus, it was selected as the optimal therapeutic approach.

## Introduction

Congenital coronary artery anomalies, encompassing variations in the origin, course, or structure of the coronary vessels, are reported in approximately 0.2-5.6% of the population, depending on the anomaly type and the diagnostic method used [1-4]. Although most cases are asymptomatic, certain variants, particularly those following an interarterial course, are associated with a greater risk of myocardial ischemia and represent the second most frequent cause of sudden cardiac death among young athletes [5-7]. Anomalous aortic origin of the right coronary artery (R-ACAOS) is a rare subtype, with an estimated prevalence of about 0.026% in Caucasian populations [4]. The clinical impact of R-ACAOS is mainly determined by its anatomical route: an interarterial trajectory between the aorta and pulmonary artery is considered high-risk due to possible vessel compression, whereas retroaortic or prepulmonic paths are usually benign [5,8]. Atrioventricular (AV) conduction abnormalities, such as first- or second-degree (Mobitz I/II) AV block, may reflect ischemia-induced conduction disturbances but can also result from age-related degenerative changes or comorbidities such as sleep apnea [9]. Advances in non-invasive imaging techniques, particularly coronary CT angiography and cardiac MRI, have substantially improved the ability to detect and characterize these anomalies [10,11]. Current evidence suggests that, even in the absence of significant ostial stenosis, an interarterial course requires careful risk assessment and, in symptomatic cases, surgical correction may be necessary to prevent adverse outcomes [12,13].

## Case presentation

Timeline of events

2019-2020

The patient is a 71-year-old male with a history of hypertension, dyslipidemia, benign prostatic hyperplasia, sleep apnea, osteoarticular problems, and vertigo. He has no history of tobacco, alcohol, or drug use. His family history includes valvular heart disease. He remained physically active after retirement as a sailor. Routine check-ups in primary care included regular ECGs and Holter monitoring.

Late 2020

The patient began experiencing mostly nocturnal palpitations and atypical left-sided chest pain, which worsened when he lay on his left side, coughed, or took deep breaths, but improved when he lay flat. There were no episodes of syncope. ECG and Holter monitoring revealed first-degree AV block, minor ischemic changes in anterolateral leads, nocturnal AV block episodes, nonspecific intraventricular conduction delays, and ST-T changes in V3-V5 at peak heart rate, suggesting possible myocardial ischemia.

2023

**Figure 1:**
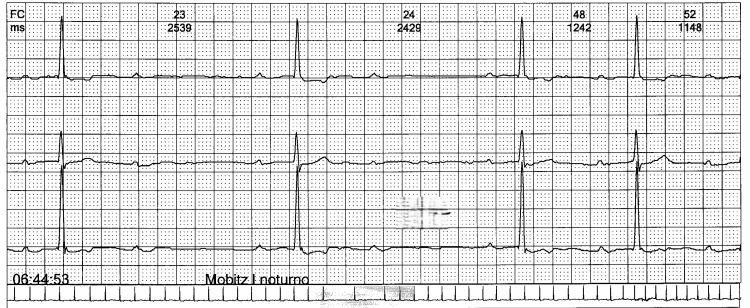
Twelve-lead ECG showing second-degree AV block, Mobitz I (Wenckebach). Progressive PR-interval prolongation until a nonconducted P wave occurs, after which the cycle repeats. Variable heart rate. Paper speed: 25 mm/s; amplitude: 10 mm/mV. AV, atrioventricular

**Figure 2:**
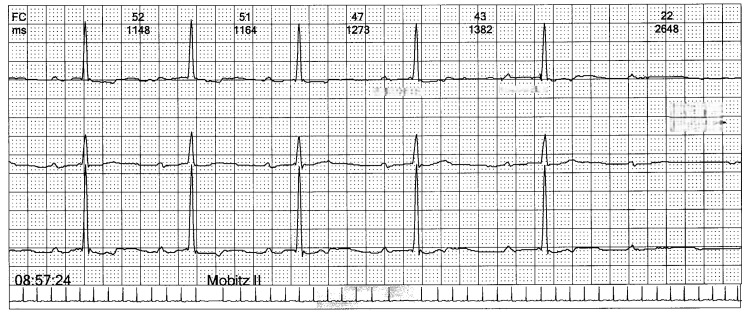
Twelve-lead ECG demonstrating second-degree AV block, Mobitz II. Sudden failure of P-wave conduction (blocked QRS) without preceding PR-interval prolongation, consistent with Mobitz II. Heart rate between 52 and 22 bpm. Paper speed: 25 mm/s; amplitude: 10 mm/mV. AV, atrioventricular

Chest pain and palpitations became more frequent. Holter monitoring showed persistent first-degree AV block, Mobitz I second-degree AV block, and nocturnal Mobitz II, but no ST-segment changes. PR intervals ranged from 220 to 240 ms, with rare dropped beats observed at night (see Figure 1 and Figure 2). Due to these findings, he was referred to cardiology for further evaluation.

2024

**Figure 3:**
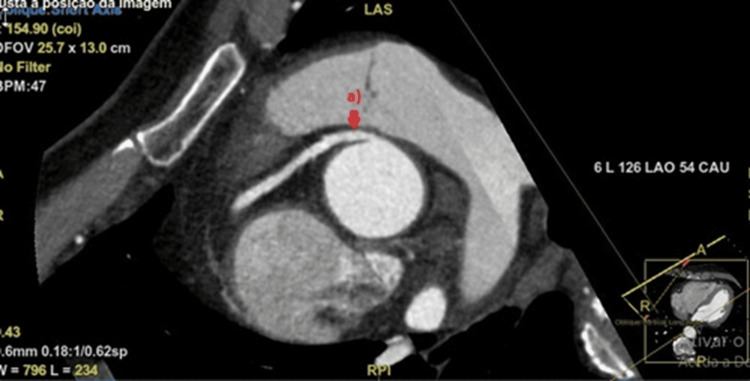
Axial slice of CCTA showing the right coronary artery (arrow) with an aortic origin at the level of the aortic wall between the right and left coronary sinuses, traveling between the aorta and pulmonary artery (interarterial course). CCTA, coronary computed tomography angiography

**Figure 4:**
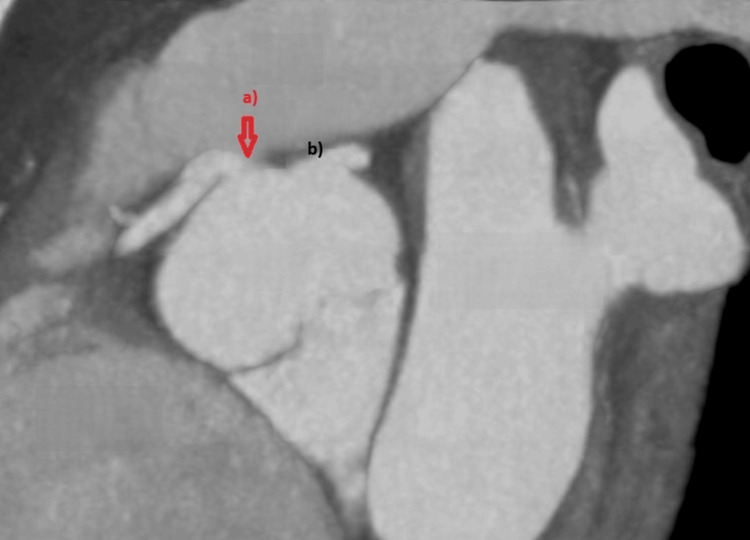
Multiplanar reconstruction of CCTA (oblique sagittal view) showing: (a) anomalous origin of the right coronary artery at the level of the aortic wall between the right and left coronary sinuses (red arrow); (b) left coronary artery origin from the left sinus. CCTA, coronary computed tomography angiography

**Figure 5:**
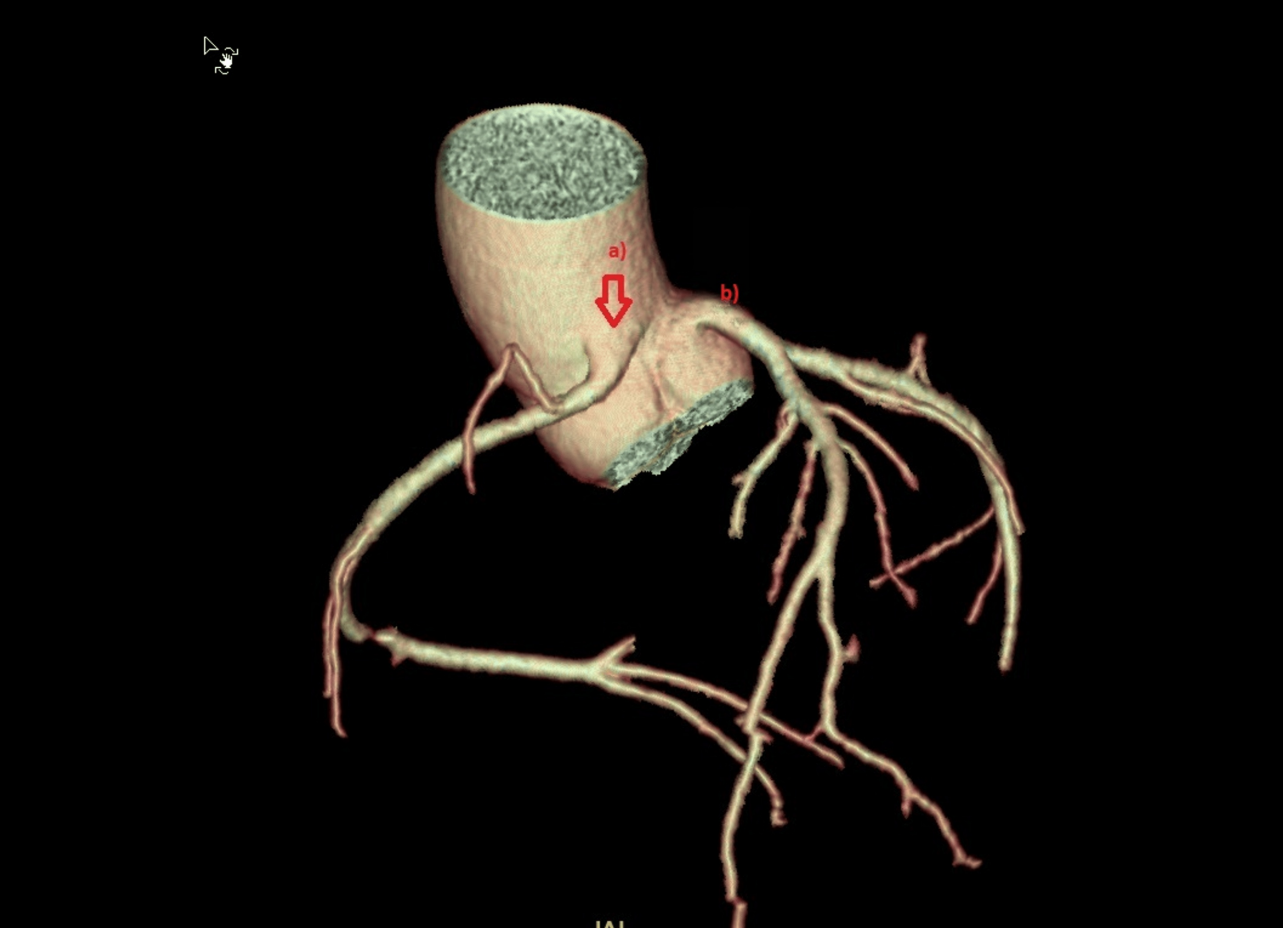
Multiplanar and 3D reconstructions of CCTA. (a) Cross-section showing the anomalous right coronary artery (arrow) arising from the aortic wall between the right and left coronary sinuses. CCTA, coronary computed tomography angiography; a, anomalous right coronary artery; b, left coronary artery origin from the left sinus

Echocardiogram showed normal biventricular function and no valve abnormalities; coronary computed tomography angiography (CCTA) showed an anomalous right coronary artery with an aortic origin between the right and left coronary sinuses, following a short interarterial course between the aorta and pulmonary artery, apparently without significant ostial narrowing, vessel caliber reduction, or other high-risk features (see Figures 3-5). Cardiac magnetic resonance (CMR) stress perfusion study showed a subendocardial defect of the inferior wall suggestive of ischemia, while the left ventricle (LV) showed normal volumes and function without evidence of fibrosis.

2025

Despite corrective measures for sleep apnea such as CPAP use, the Holter monitor test was repeated, and Mobitz I and II levels remained unchanged; an exercise stress test was not performed, as symptoms were reproducible at rest and occurred at night, and CMR was suggestive of ischemia.

Management and outcome

Given the presence of symptoms (atypical chest pain and palpitations), conduction abnormalities, inferior wall ischemia, and the interarterial course of the right coronary artery, a multidisciplinary team (cardiology and cardiothoracic surgery) recommended surgical unroofing [12-14]. Other options, such as conservative management or percutaneous intervention, were considered less suitable due to ongoing symptoms and anatomical risks [12]. The patient was scheduled for surgical unroofing with expectations of resolving chest pain and palpitations and normalizing AV conduction and any residual ischemia [13,14].

Patient perspective

The patient expressed frustration at the initial delays in diagnosis but felt relieved once the cause was identified. He was pleased with the possible outcome of the surgery and grateful for the coordinated care and clear communication throughout the process.

## Discussion

This case demonstrates the diagnostic and therapeutic challenges of R-ACAOS with an interarterial path in an older adult [1,5,8]. The patient’s symptoms, nocturnal palpitations and atypical chest discomfort, were initially vague but, combined with conduction disturbances and cardiovascular risk factors, warranted deeper investigation [9]. The timing of nocturnal symptoms and conduction blocks suggests transient ischemia of the conduction system, possibly due to dynamic compression of the anomalous artery, especially during shifts in intrathoracic pressure (such as lying on the left side or sleep apnea) [15-17]. Nonetheless, other causes for AV block, like degenerative disease or sleep apnea, were also considered [9,15,16]. Precise anatomical characterization is crucial, as an interarterial route carries a higher risk of ischemia and sudden death, particularly during exertion or sympathetic activation [6,7,17]. Management options include observation, medical therapy, percutaneous intervention, or surgery [12,14]. In this case, despite CT imaging revealing a short interarterial course without high-risk features, the presence of symptoms (atypical chest pain and palpitations) and CMR confirmation of inferior wall ischemia supported the decision for surgical intervention [1,10-12,18-20].

Recent advances in the diagnosis and management of coronary artery anomalies have emphasized the importance of integrating clinical symptoms, functional data, and detailed anatomical assessment using CCTA and CMR [21]. As highlighted in the literature [21], therapeutic decisions should be individualized, considering both anatomical findings and evidence of ischemia or persistent symptoms.

Given the patient’s persistent symptoms and inferior wall ischemia, surgical unroofing was preferred, as it is associated with excellent outcomes and low perioperative risk in selected patients [9,10,12,14]. Recent studies show symptom resolution in over 90% of cases and minimal complications [13]. Early recognition of atypical symptoms and continued surveillance in primary care were key. Ultimately, multidisciplinary evaluation and tailored management are essential in these rare scenarios [12,21].

## Conclusions

This case highlights the importance of considering multifactorial causes, such as sleep apnea and coronary artery anomalies, even short interarterial courses without high-risk features, in patients with unexplained chest pain and conduction system disturbances, including the elderly. Detailed anatomical assessment and correlation with clinical symptoms are essential for appropriate management. In these cases, a detailed anatomical assessment (e.g., with CCTA and CMR) can be fundamental to understanding the clinical context and guiding therapeutic decisions. The correlation between imaging findings and symptoms is what determines the management and follow-up plan for the patient.

Additionally, it is important to emphasize that sleep apnea is often an underestimated factor, yet it has a significant impact on arrhythmias and cardiovascular symptoms. This differential diagnosis should always be considered, particularly in the presence of suggestive symptoms such as daytime sleepiness or resistant hypertension. Multidisciplinary evaluation and tailored management are essential in these rare scenarios. The patient is awaiting surgical unroofing. According to published series, this approach is associated with high rates of symptom resolution and low perioperative risk; outcomes in this case will be monitored accordingly. This case underscores the crucial role of coordinated care between primary and specialized services.
